# Robotics and Artificial Intelligence and Their Impact on the Diagnosis and Treatment of Cardiovascular Diseases

**DOI:** 10.7759/cureus.42252

**Published:** 2023-07-21

**Authors:** Abdullah Saeed, Abdullah AlShafea, Abdulrahman Bin Saeed, Maliha Nasser, Rihana Ali

**Affiliations:** 1 Research Unit, Ministry of Health, Abha, SAU; 2 Public Health, King AbdulAziz University, Khamis Mushayt, SAU

**Keywords:** cardio vascular surgery, preventive, cardio vascular disease, ai and robotics in healthcare, ai & robotics in healthcare

## Abstract

A new era has begun in the treatment of cardiovascular disorders as a direct result of the significant developments that have been made in robotics and artificial intelligence (AI). This abstract investigates the potential and impact that AI algorithms and robotic systems may have in the diagnosis and treatment of cardiovascular problems. The field of cardiovascular treatments has been completely transformed by robotically assisted surgeries, which have enabled minimally invasive procedures with increased patient safety and decreased recovery time. The incorporation of AI algorithms into cardiovascular care has made early abnormality identification, risk classification, and tailored treatment planning significantly easier. However, problems including patient safety, data privacy, and smooth integration into existing healthcare systems need to be solved. This abstract places an emphasis on the necessity of collaboration and responsible implementation in order to fully harness the promise of robotics and AI in cardiovascular care, which will ultimately lead to improved patient outcomes and an enhanced quality of life.

## Editorial

We are writing to offer our ideas and insights regarding the incredible achievements in the field of robotics and artificial intelligence (AI), as well as the impact that these advancements have had on the detection and treatment of cardiovascular disorders. We hope you find this to be an interesting and thought-provoking read. As we continue to witness unimaginable leaps forward in the field of medical technology, it is becoming increasingly important to investigate and assess the possibilities of new medical technologies in the fight against one of the main causes of death around the world: cardiovascular diseases.

The prevention and treatment of cardiovascular illnesses, which include ailments such as heart attacks, strokes, and heart failure, present substantial difficulties to medical practitioners all around the world. The burden of chronic diseases is only likely to increase further as a result of the increasing prevalence of sedentary lifestyles and the aging of the population [1]. The application of robotic systems and artificial intelligence algorithms within the field of cardiovascular medicine, on the other hand, holds the potential to revolutionize patient care while enhancing outcomes. The use of robotic technology has already established itself in a number of subspecialties of medicine, where it provides a level of precision and dexterity that is unmatched in surgical procedures. Robotics has emerged as a transformative force in the field of cardiovascular therapies, making it possible to perform minimally invasive procedures that reduce the amount of patient trauma, shorten the amount of time needed for recovery, and improve overall patient safety. It has been established that robotic-assisted operations, such as robotically assisted coronary artery bypass grafting (CABG) and robotically-assisted percutaneous coronary interventions (PCI), yield exceptional results, and they continue to develop as new technologies become available [2]. In addition, the integration of AI algorithms into cardiovascular care has opened up new avenues for the diagnosis of disease, the classification of risk, and the formulation of treatment strategies. AI-driven diagnostic tools can evaluate medical pictures, electrocardiograms, and patient data with a speed and precision that is unmatched by traditional methods. These tools were trained on enormous datasets. This allows for the early detection of cardiovascular irregularities and helps in forecasting the course of the disease as well as the patient's response to treatment. In addition, AI algorithms have the ability to assist medical personnel in improving patient outcomes by means of personalized medicine approaches, optimizing treatment techniques, and individualizing medications [3].

It is essential to acknowledge the obstacles and address any issues related to the introduction of robotic systems and AI in cardiovascular care. While the utilization of these technologies brings a number of opportunities, it is essential to acknowledge the challenges and address any concerns. Important concerns that need to be thoroughly examined and handled include preserving the confidentiality and security of patient information, protecting the privacy of patients' medical records, and ensuring that these technologies can be effectively integrated into the existing healthcare infrastructure [4]. It is necessary for healthcare practitioners, academics, and policymakers to participate in vigorous debates and collaborate in order to harness the full potential of robotics and AI in the management of cardiovascular disease in this fast-shifting landscape. We can pave the way for a future in which cardiovascular illnesses will be more effectively prevented, identified, and treated, which will ultimately lead to improved patient outcomes and an enhanced quality of life if we accept these technological breakthroughs in a responsible and ethical manner [5].

**Figure 1:**
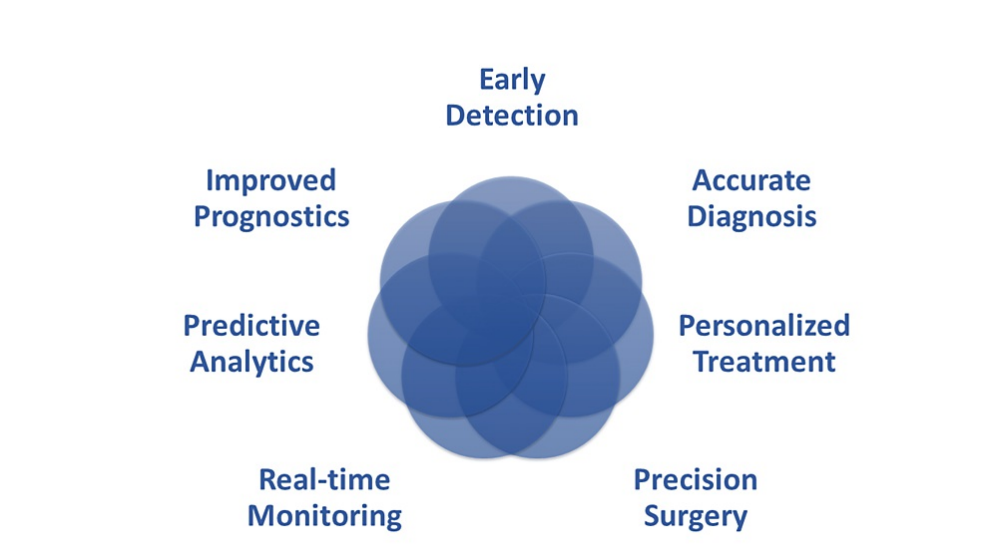
Advantages of Robotics and Artificial Intelligence (AI) in Diagnosis and Treatment of Cardiovascular Diseases

**Figure 2:**
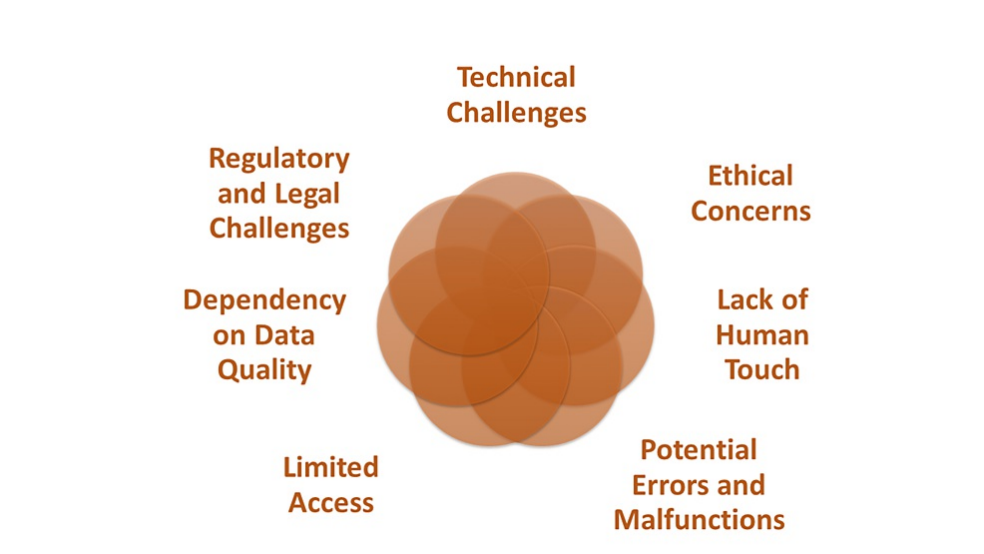
Disadvantages of Robotics and Artificial Intelligence (AI) in Diagnosis and Treatment of Cardiovascular Diseases

We will continue to look into specific applications of robots and AI in cardiovascular illness, highlight recent developments, and evaluate the challenges and opportunities that lie ahead. Figures 1, 2 summarize the advantages and disadvantages of the use of robotic and AI technology in cardiology. We believe that by putting light on these themes, we can stimulate additional study and innovation in the field of cardiovascular medicine as well as develop a greater knowledge of the possibilities of these transformative technologies. Preserving data in healthcare settings for robotics and AI in diagnosing and treating cardiovascular diseases in developing countries requires robust security measures, standardized data collection, reliable storage infrastructure, collaborative data sharing, ethical considerations, training, supportive regulations, local infrastructure development, and continuous monitoring and evaluation.
